# Predicting Hemodynamic Shock from Thermal Images using Machine Learning

**DOI:** 10.1038/s41598-018-36586-8

**Published:** 2019-01-14

**Authors:** Aditya Nagori, Lovedeep Singh Dhingra, Ambika Bhatnagar, Rakesh Lodha, Tavpritesh Sethi

**Affiliations:** 1 CSIR-Institute of Genomics and Integrative Biology, New Delhi, 110007 India; 2 Academy of Scientific and Innovative Research (AcSIR), Ghaziabad, 201002 India; 3 All India Institute of Medical Sciences, Department of Pediatrics, New Delhi, 110029 India; 4 Indraprastha Institute of Information Technology Delhi, 110020 Delhi, India; 5 Stanford University, School of Medicine, Stanford, 94305 CA USA

## Abstract

Proactive detection of hemodynamic shock can prevent organ failure and save lives. Thermal imaging is a non-invasive, non-contact modality to capture body surface temperature with the potential to reveal underlying perfusion disturbance in shock. In this study, we automate early detection and prediction of shock using machine learning upon thermal images obtained in a pediatric intensive care unit of a tertiary care hospital. 539 images were recorded out of which 253 had concomitant measurement of continuous intra-arterial blood pressure, the gold standard for shock monitoring. Histogram of oriented gradient features were used for machine learning based region-of-interest segmentation that achieved 96% agreement with a human expert. The segmented center-to-periphery difference along with pulse rate was used in longitudinal prediction of shock at 0, 3, 6 and 12 hours using a generalized linear mixed-effects model. The model achieved a mean area under the receiver operating characteristic curve of 75% at 0 hours (classification), 77% at 3 hours (prediction) and 69% at 12 hours (prediction) respectively. Since hemodynamic shock associated with critical illness and infectious epidemics such as Dengue is often fatal, our model demonstrates an affordable, non-invasive, non-contact and tele-diagnostic decision support system for its reliable detection and prediction.

## Introduction

Shock is a clinical state of mismatch between the demand and supply of cellular oxygen. It is most commonly associated with fluid loss (hypovolemia), inefficient pumping (cardiogenic) or infections (septic) that cause redistribution of fluids within the body. All three mechanisms contribute, often in combination, to shock in the intensive care unit (ICU). Shock affects almost 30% of ICU patients^1^ with mortality rates as high as 34% especially in the developing countries^2^. Although reversible in initial stages, delay in detection leading to ineffective management of shock often leads to rapid deterioration of tissue function, failing organs (lungs, liver, kidney, gut) and eventually death. The 2014 consensus update on shock has recommended a more aggressive approach to hemodynamic assessment including response to fluids and sequential evaluations^3^. Aggressive management that has been shown to improve outcomes^4–6^. While continuous assessment is vital, many of the recommended procedures involve invasive monitoring which substantially contributes to hospital-acquired infections. The non-invasive methods, such as cuff-based BP monitoring and ultrasonography are not continuous and additionally require skin-contact which is a risk factor for infections especially in neonates and infants^7^. Importantly, hemodynamic compromise is not only prevalent in the ICUs but also in the community in developing countries with low doctor-to-patient ratio and high rate of infections such as dengue and diarrhea that often lead to shock. Hence, a non-invasive, non-contact modality for monitoring hemodynamic status is highly desirable for guiding shock management.

**Figure 1:**
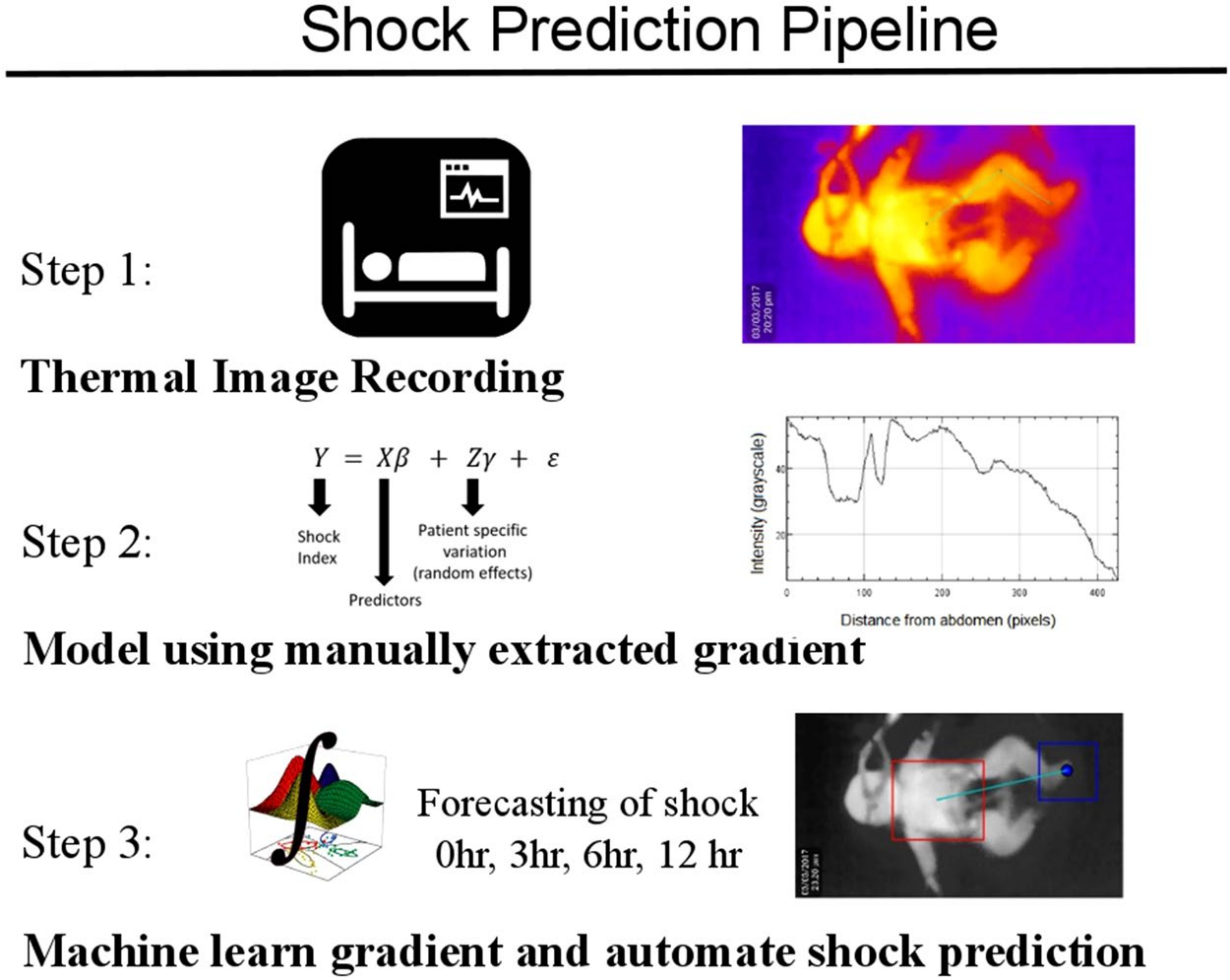
Summary of the pipeline used for automating shock prediction. Step 1 shows capture of vitals and thermal images. Step 2 shows the generalized mixed modeling approach to validate the hypothesis using manually extracted gradient (right). Manual extraction of CPD was done using an image processing software FIJI^18^. Most images show a negative linear gradient i.e. higher thermal intensity of abdomen than foot. The early local dip (U-shaped) corresponds to diaper and may be ignored. Step 3 shows the machine learning based region of interest segmentation followed by prediction of shock at 0, 3, 6 and 12 hours.

Here we report a non-invasive, non-contact modality constructed using a combination of thermal-imaging, machine-learning and longitudinal data analysis for detection and prediction of shock in patients admitted to a pediatric intensive care unit (PICU). We had earlier reported the feasibility of such recordings on a small number of images by using an affordable device attached to Android Smartphone^8^ and its potential value for shock-detection using a hand-calculated feature, central-to-peripheral temperature difference, a known feature of shock^9,10^. In this paper, we expand the feasibility study to detect and predict (forecast) shock up to 12 hours in advance using a fully automated computer vision and machine-learning pipeline (Fig. 1). We do this by training random forest classifiers for ROI extraction using shape features extracted from thermal images. Shock-index, defined as the ratio of heart rate and systolic blood pressure is one of the important measures of hemodynamic instability^11,12^ and guided the model learning as an outcome variable. A longitudinal data modelling approach using generalized linear mixed-effects model for detection and prediction of binary shock-index is tested. Hence the aim of this study was to construct a robust, non-invasive, non-contact, automated and affordable pipeline for shock prediction using machine learning and this is demonstrated through superior model prediction indices. The potential to scale beyond the intensive care settings to emergency rooms and the community make this study especially valuable for decision making for shock in resource-limited settings.

## Results

### Patient demographics

Cohort characteristics and statistical differences in shock versus non-shock groups are shown in Table 1. Shock and Non-shock were defined on the basis of Shock Index Pediatric Age-adjusted calculation. As expected, pulse rate (and heart rate) showed the most significant difference between the two groups, followed by age.

**Table 1:** Cohort characteristics of the control (no-shock) and affected (shock) group. p-values for difference were computed using Wilcoxon (W) rank sum test (non-parametric) or Student’s t-test (t) (parametric) after testing for the normality assumption.

Variable	Non-shock Images *n* = *147*	Shock Images *n* = *106*	Statistical Test
	Mean (SD)	Mean (SD)	p-value (W/t)
Age (months)	33.23 (46.76)	58.33 (53.84)	5.97E-07 (W)
Mean Arterial Blood pressure, mm Hg	71.66 (19.33)	63.45 (16.69)	0.002162 (W)
Arterial Systolic Blood pressure, mm Hg	97.55 (21.81)	88.97 (21.62)	0.004605 (W)
Arterial Diastolic Blood pressure, mm Hg	57.64 (16.63)	50.55 (14.87)	0.000935 (W)
Heart rate, per min	120.75 (24.53)	136.16 (22.78)	5.83E-07 (t)
Respiratory rate, per min	34.14 (14.12)	33.43 (17.96)	0.289486 (W)
Oxygen saturation (SpO2)%	92.26 (9.16)	92.79 (8.48)	0.852786 (W)
Pulse Rate, per min	118.65 (24.55)	135.91 (23.03)	4.78E-08 (t)
Shock Index	1.32 (0.44)	1.63 (0.52)	1.03E-05 (W)
Abdomen Intensity	164.24 (21.53)	161.82 (17.73)	0.69571 (W)
Foot Intensity	144.69 (20.16)	136.43 (24.16)	0.003386 (W)
Difference between pixel intensities of abdomen and foot	19.56 (23.48)	25.39 (28.88)	0.042471 (W)

### Center-to-periphery difference achieved 75% accuracy in detecting and predicting shock

**Figure 2:**
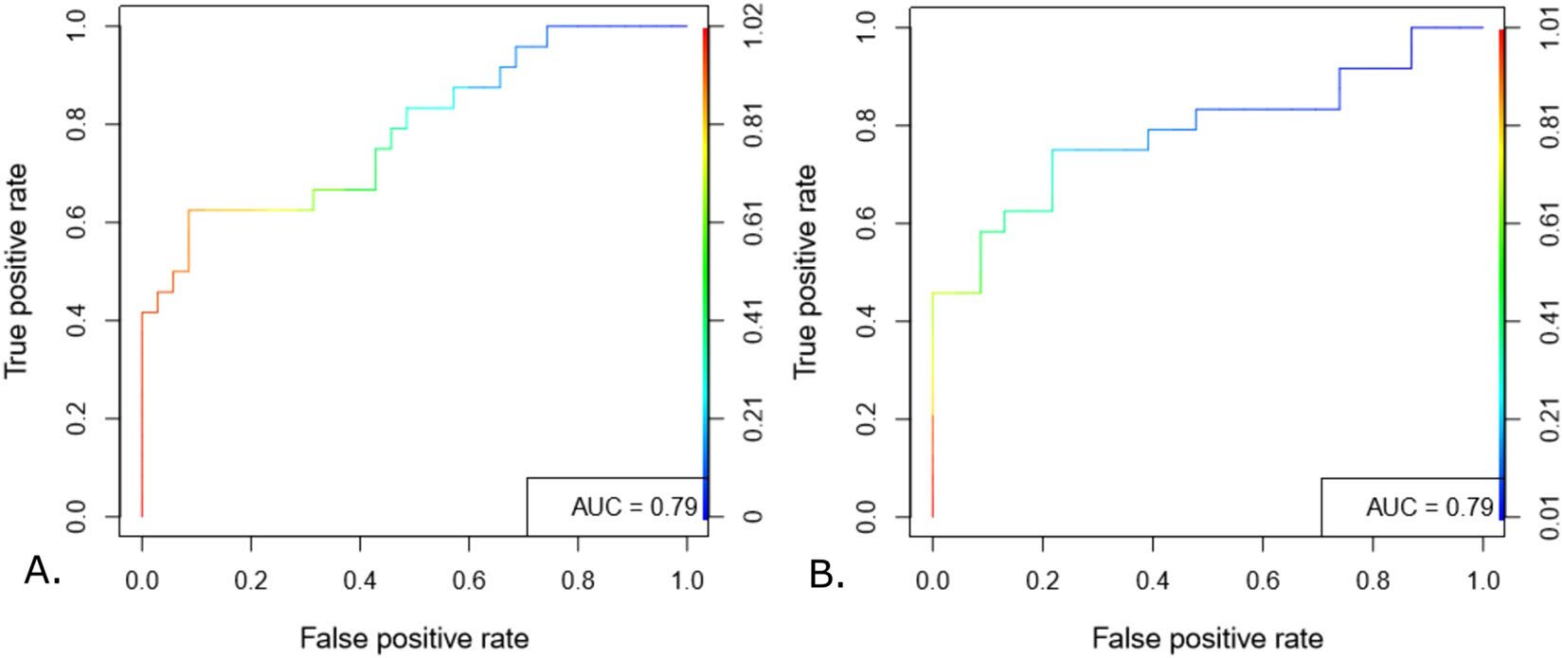
Validation of manually derived CPD for shock detection and prediction. Generalized linear mixed effects models were constructed through a standard training-testing partition. ROC curves constructed on the predictions obtained on testing set achieved 79% AUC for 0 hr detection (**A**) and 79% AUC for 3 hr prediction (**B**). ROC curves for 6 hr and 12 hr prediction are shown in Supplementary Fig. S4.

A representative thermal image with manual extraction of center-to-periphery difference (CPD) is shown in Fig. 1 (top-right), clearly demonstrating a difference in intensity between abdomen and foot (open dots). CPD was calculated along the lines (green) joining the abdomen and foot and was used as a predictor in the generalized mixed model for detection and prediction of binary shock index (response variable). The non-linear, U-shaped dip present in most images corresponds to the diaper worn by the children and was ignored. Figure 2 shows the area under the ROC curve for detection (0 hr) and prediction (3 hr) generalized linear mixed effects models. A test set accuracy of 75% in detection and 3 hr prediction were achieved (Table 2. AUC = 79%, sensitivity = 0.69, specificity = 0.79), thus validating the non-contact, non-invasive potential for CPD. Similar accuracies were obtained at 3 hr, 6 hr and 12 hr post-imaging models (Table 2). This result encouraged us to construct automated pipelines for shock detection and prediction without minimal manual intervention as a next step.

**Table 2:** Performance of Model predicting binary shock index using manually calculated CPD.(Coeff—Model coefficient of difference percent, AUC—Area Under the curve, PPV—Positive predictive value, NPV—Negative predictive value, Cut-off—Average Threshold of Ten Folds, NS- Non-shock, S- Shock).

Time-point, (NS, S)	Coeff	AUC	Accuracy	Sensitivity	Specificity	PPV	NPV	Cut-off
		Mean(SE)	Mean(SE)	Mean(SE)	Mean(SE)	Mean(SE)	Mean(SE)	Mean
0 hr (detection), (146, 107)	0.03	0.79 (0.02)	0.75(0.02)	0.69(0.05)	0.79(0.05)	0.72(0.05)	0.8(0.03)	0.47
3 hr (prediction), (141, 107)	0.01	0.79(0.04)	0.74(0.04)	0.72(0.05)	0.78(0.07)	0.73(0.06)	0.81(0.03))	0.45
6 hr (prediction), ((129, 109))	0.01	0.65(0.04)	0.66(0.02)	0.48(0.09)	0.81(0.06)	0.72(0.07)	0.71(0.05)	0.62
12 hr (prediction), (131, 118)	0.01	0.7(0.03)	0.69(0.02)	0.67(0.03)	0.68(0.04)	0.65(0.06)	0.7(0.03)	0.41

### Histogram of oriented gradients combined with random forest achieved 99% and 94% AUC for abdomen and foot segmentation

**Figure 3:**
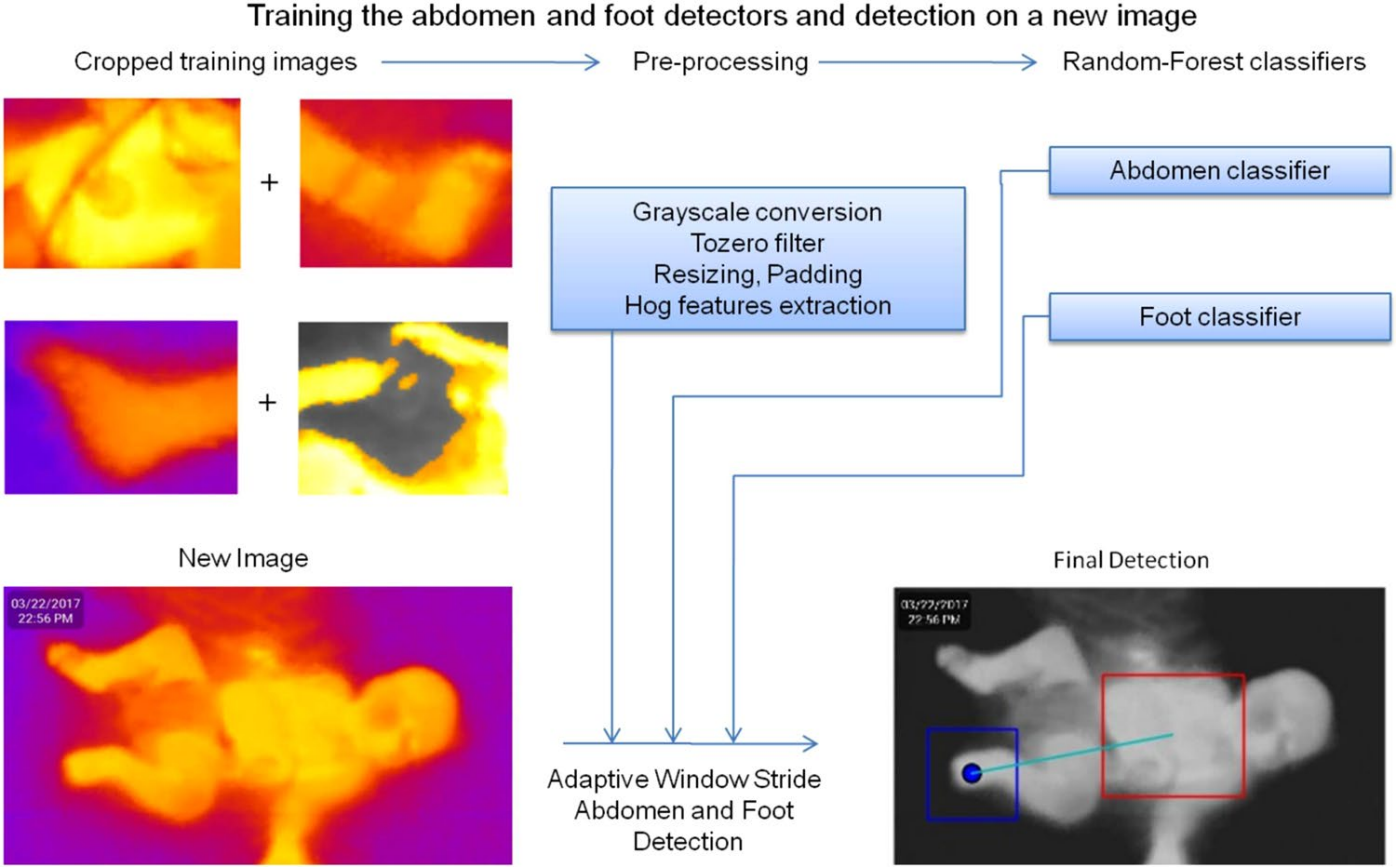
Illustration of steps in the automated detection of abdomen and foot ROIs using Random Forest based classifier on HOG features. Each classifier was trained using positive (e.g. foot) and negative (e.g. not foot) samples and adaptive window sizes were slid over the image to achieve scale-invariance of detection. Median of intensity over the detected ROIs was taken and their difference was used as a predictor (CPD). Relative CPD (percent normalized to abdomen) was used to make the feature more robust to ambient thermal variations.

**Figure 4:**
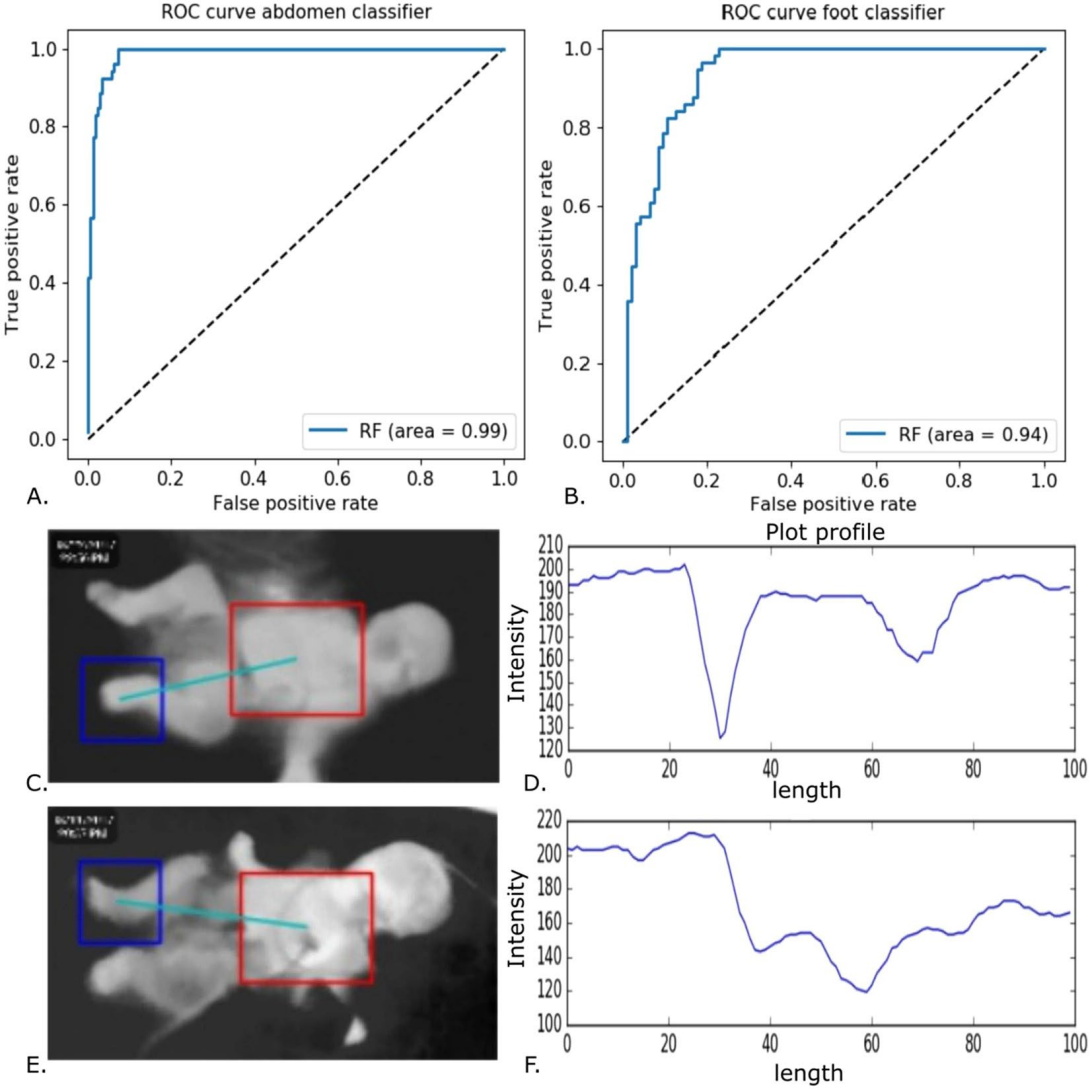
Quality evaluation of detected foot and abdomen using the computer-vision automated-detection pipeline. Area under the ROC curve of 99% was achieved for the optimized abdomen classifier and 94% was achieved for the optimized foot classifier. Bounding boxes (**C**,**E**) for foot and abdomen in a non-shock and shock patient were constructed by the algorithm. The intensity gradient along the centers of these (**D**,**F**) recapitulated the manual gradient shown in Fig. 1, albeit without any manual intervention and was taken forward for longitudinal modeling.

Thermal images are often noisy as the IR radiation is diffuse. Thus, these lack sharp features and may be contaminated by ambient thermal noise. Since we could not use texture or color for recognition of ROIs, a pipeline relying upon shape features was designed. Figure 3 illustrates the steps of this pipeline which included image processing, HOG feature generation and Random Forest classifiers for foot and abdomen detection. The classifiers constructed a bounding box and an ROI (lower right), which were then evaluated against the human-labeled ground truth. Scale-invariance of detection was achieved by using an adaptive window size while detecting the foot and abdomen, with a resultant AUC of 99% for abdomen detection and 94% for foot detection evaluated against the human annotated ground truth (Fig. 4. The median intensity of the ROIs were taken forward for constructing longitudinal models for detection and prediction of shock. Topical subheadings are allowed.

### Shock detection using automated difference

The ROI’s around foot and abdomen were used for further automation of shock detection using the automated CPD. Generalized mixed effects model using repeated patient samples as random effects and age, pulse rate as covariates achieved 0 hr detection accuracy of 73% (sensitivity = 0.58, specificity = 0.81), Table 3. The ROC curve for automated shock detection is shown in Fig. 4A. Since our model does not include the prior history of shock status in a patient, it can be used for classification of shock/no-shock from a snapshot alone.

**Table 3:** Performance of Model predicting binary shock index using automated detection of CPD.(Coeff—Model coefficient of difference percent, AUC—Area Under the curve, PPV—Positive predictive value, NPV—Negative predictive value, Cut-off—Average Threshold of Ten Folds, NS - Non-Shock, S- Shock).

Time-point, (NS, S)	Coeff	AUC	Accuracy	Sensitivity	Specificity	PPV	NPV	Cut-off
		Mean(SE)	Mean(SE)	Mean(SE)	Mean(SE)	Mean(SE)	Mean(SE)	Mean
0 hr, (119,84)	0.03	0.75 (0.03)	0.73(0.03)	0.58(0.06)	0.81(0.06)	0.74(0.05)	0.75(0.03)	0.55
3 hr, (108,86)	0.02	0.77(0.04)	0.73(0.03)	0.65(0.06)	0.82(0.03)	0.75(0.04)	0.74(0.05)	0.49
6 hr, (100,88)	0.002	0.68 (0.03)	0.69 (0.02)	0.58(0.07)	0.74(0.06)	0.79(0.04)	0.64(0.02)	0.51
12 hr, (101,97)	0.01	0.69(0.04)	0.67(0.03)	0.62(0.07)	0.73(0.07)	0.72(0.05)	0.69(0.04)	0.48

### Shock prediction using automated difference

**Figure 5:**
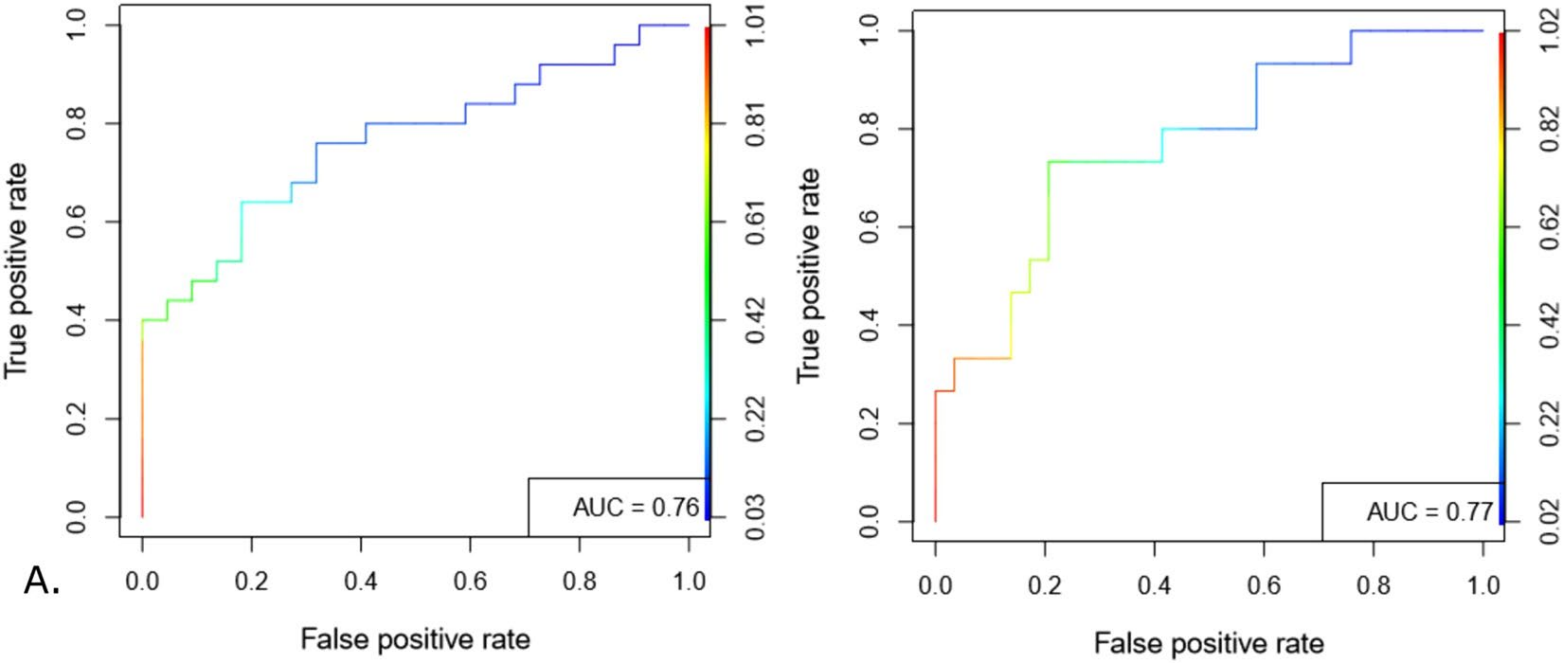
ROC curves for 0 hr detection and prediction models achieved reasonably good AUC. 0 hr time-point (**A**) shock-index detection model based on automated-detected difference showed an AUC of 76% and the 3 hr (**B**) shock-prediction model based on detected difference showed a similar AUC of 77%. Results from models for 6 hr and 12 hr are shown in Table 3. ROC curves for 6 hr and 12 hr prediction are shown in Supplementary Fig. S5.

The SAFE-ICU resource allowed us to mine time-stamped shock indices at 3 hr, 6 hr and 12 hr from the time of imaging. Similar models using mixed effects were constructed for these time points as well to evaluate the predictive potential of automated CPD. Interestingly, the models were able to predict the binary shock-index status, especially at 3 hours from imaging with an accuracy of 73% (PPV 75%, NPV 74%) (Table 3, Fig. 5B). In an ICU where patients may rapidly decompensate and end up in failure, a lead time of up to 3 hours can make a considerable difference in saving lives and in preventing organ failure which contributes to major human and economic morbidity in ICU survivors. Since the models were evaluated upon 10 repeated partitions, we could assess and compare the stability of AUC between manual and automated shock prediction models (Supplementary Figure S1). As expected, the manual and automated AUC’s follow each other closely. The manual models performed slightly better at 0 hr and 3 hr whereas the automated pipeline demonstrated slightly superior performance at 6 hr and 12 hr of shock prediction. In this result as well, the model was agnostic to prior history of the patient’s shock status, hence useful for forecasting of shock using a snapshot alone, without the need for capturing repeated images.

## Discussion

This study presents a machine-learning algorithm trained upon thermal images for continuous, non-contact detection and prediction of shock in the PICU. Resource-constrained settings motivated the use of an affordable and compact thermal array coupled to an Android smartphone, thus ensuring hardware scalability and potential for ER and community use beyond ICUs. Further, we intentionally kept the models lean to avoid the need for a graphics processing unit (GPU), making our study especially relevant for developing countries with low doctor-to-patient ratio and lack of skilled professionals. Artificial intelligence and machine-learning are expected to disrupt healthcare and bring novel understanding to disease mechanisms and delivery of healthcare. Several recent studies have shown that advanced artificial intelligence methods such as deep-learning can out-perform doctors at specific tasks such as skin cancer^13^, arrhythmias^14^ and retinal disease^15^ detection. However, we took an alternative approach to leverage physiological hypothesis in order to keep the hardware and models lean. Body surface thermal gradients are known to exist in shock and we developed the machine learning pipeline around it. Skin-surface is a key area for evaluating the perfusion status of body as its temperature is correlated with blood flow^16^. However, the potential for surface body temperature patterns using machine learning and computer vision for hemodynamic status has been under-explored. Most common sensors available in the ICUs measure temperature at a single point and fail leverage temperature gradients. Our study demonstrates that affordable and compact thermal imaging can effectively capture the CPD gradient that may inform shock diagnosis and prediction. Since manual annotations are expensive, repetitive, time-consuming and a hindrance to automation, our computer vision based machine learning pipeline classified the key central and peripheral areas in the human body. The recording of images needed minimal training or technical expertise, limited to handling the imager and taking precautions for reliable capture. Relative CPD percent added robustness against ambient thermal noise. We experimented with a wide range of features based upon intensity statistics, clustering and shape and found the shape-tailored features (HOG) to be a good fit for ROI detection in thermal images. Since machine learning algorithms are not yet mature for handling non-uniformly sampled longitudinal data, we applied generalized linear mixed effects models to leverage and account for repeated images from same subjects. Such models have been extensively used in statistical analysis of longitudinal clinical studies^17^. In an earlier study^8^, we had shown the correlation between lactate levels and the manually calculated CPD. However, in this study, we restricted ourselves to the most easily obtainable features such as age and pulse rate with the goal of non-invasive, continuous and scalable monitoring to ICUs, ERs and community health settings. Our models achieved reasonably useful (75% or higher) positive predictive and negative predictive values, the key indicators for clinical applicability for a machine learning model. AUROC, accuracy, sensitivity and specificity also demonstrated good performance of our models, nearly reaching the manual modeling of shock using CPD. We note that our model is generally more specific than sensitive and may have some value for ruling out shock. However, an omission of action in suspected shock, and critical care settings in general, may have drastic consequences, hence we do not advocate the use of our model for omission of fluid management decisions. Further studies on a larger scale may be necessary to explore the use in decisions involving fine-tuning of fluid management in shock. The expansion of our automated detection and prediction of shock beyond the intensive care and emergency room settings may be facilitated in the developing countries through rural support mechanisms such as the Accredited Social Health Activist (ASHA) workers, women who promote and educate health in the local rural Indian settings. With this end in mind, our model does not require prior history, thus able to classify and predict (forecast) shock from a snapshot in our settings. The results from our study encourage us to believe that lean development with state-of-the-art technology may be the most feasible solution for the developing world with low doctor-to-patient ratio and high disease prevalence. All our data and code for modeling are made openly available as per FAIR (findable, accessible, inter-operable and reusable) research guidelines (see Data availability section).

The current study has a few limitations. It was conducted on a small number of patients, owing to the requirement for the gold standard measurement of intra-arterial invasive BP monitoring. This was extracted from the ***SAFE-ICU*** resource that has been warehousing vitals, demographics, laboratory investigations and treatment charts since early 2016. In order to salvage more patient numbers, we tested whether the non-invasive BP can be used as a proxy for intra-arterial BP by constructing a model that predicts latter from the former. However, as per expectation, the intra-arterial BP had a consistent positive offset (intercept) and the model did not explain sufficient variance (*R*^2^) to be useful (Supplementary Table S1). The other major limitation of this study is the lack of multiple clinical sites to validate the generalizability of the models. Ongoing work and partnerships with clinical and community organizations will address this limitation in future and the tele-diagnostic potential of the modality will be combined with models deployed on the cloud to offer decision support where adequate clinical expertise is not available. In conclusion, this study demonstrates the value of using thermal imaging, machine learning and generalized mixed effects models in conjunction for detection and prediction of shock with high AUC (76%) up to 3 hours and with a reasonable AUC (70%) up to 12 hours. This opens up a important window of opportunity to initiate early interventions such as fluid and vasopressor management before the downward spiral of irreversible organ damage sets in.

## Methods

### Cohort and Study Design

The study was carried out at the Pediatric Intensive Care Unit of All India Institute of Medical Sciences, a tertiary care hospital in New Delhi, India. Since thermal images only capture infra-red radiation, these don’t reveal patient identity and study did not involve any contact or change in routine patient care. Hence a waiver of consent was sought and granted by the Institute Ethics Committee (Ref. No. IEC/NP-211/08.05.2015, AA-2/09.02.2017). The period of the study was from May 2016 - September 2016 and from February 2017 - April 2017. Data were collected on all patients admitted during this duration (patients enrolled) and further limited to include only patients with Arterial blood pressure recording (patients analysed). This was deliberately enforced to train models against the gold standard for hemodynamic evaluation. The study design was prospective longitudinal since multiple time points from each patient were recorded. A comparison of baseline characteristics in the shock versus non-shock group was evaluated using a two-sample two-tailed Student’s t-test or a Wilcoxon’s rank sum test after checking for the assumption of normality using Shapiro Wilk’s test for normality.

### Thermal Imaging

A standard operating procedure was followed for the recording of thermal images in order to minimize the effect of extraneous factors such as device handling, patient positioning, environment etc (Supplementary Methods S1). Briefly, it was ensured that the ambient temperature was comfortable and the patient was uncovered. Thermal images were clicked in a standard color-scale ensuring that the full body (in infants) or at least abdomen and feet were exposed. A total of 253 thermal images from 51 children (27 male, 24 female), ages ranged between 0.2–144 months and with arterial line recordings available, were recorded using a commercially available Android Smartphone attachment (Seek Thermal®). Images were acquired at different time points (i.e. on different days), therefore, the same patient could have different values for shock-status on different days which eliminates bias due to patient’s propensity characteristics (e.g. age and gender). An average of five images was recorded for each child. Vitals corresponding to the time-stamp of image were extracted from the data warehoused at 15 second intervals (***SAFE-ICU***^8^).

### Manual extraction of the Central-to-peripheral difference in intensity

Central-to-peripheral Difference (CPD) was manually extracted using FIJI^18^, an image processing software platform. CPD difference percentage (diff-percent) was calculated as follows,

$$Difference\,percentage=\frac{Abdomen\,Intensity-Foot\,Intensity}{Abdomen\,Intensity}\ast 100$$

### Automated extraction of the Central-to-peripheral difference in intensity

The original Red-Green-Blue (RGB) thermal images were (1108, 624, 3) dimensional matrices which were analysed using an in-house computer-vision based pipeline (Fig. 3) written in Python^19^ using OpenCV^20^ version 3 library. Abdomen/non-abdomen and foot/non-foot areas within images were cropped for training the classifiers. Images were converted from RGB to grayscale. Pixel intensities were thresholded by the median of each image in order to remove thermal noise. Contrast-limited adaptive histogram equalizer (CLAHE) was used to enhance contrast for better detection of colder feet. Abdomen and foot images were padded and resized to (50, 50) and (100, 100). Images were partitioned into a 70%-30% training and test sets. Histogram of oriented gradients^21^ (HOG) features were extracted and random forest^22^ classifiers were constructed with 310 trees for abdomen and 160 trees for foot (optimized using out-of-box error) using library sklearn^23^. The learned classifiers were evaluated on test set and used for detecting abdomen and foot in thermal images. To account for variation in the size of foot/abdomen, an adaptive-size window proportional to the full body silhouette was used (Fig. 3). Median intensities of the adaptive windows in foot and abdomen were calculated to avoid the influence of outliers (e.g. tubes). Finally, the detected difference between median intensities expressed as a percentage was used as the automated CPD feature for further modeling.

$$Detected\,difference\,percentage=\frac{Detected\,Abdomen\,Intensity-Detected\,Foot\,Intensity}{Abdomen\,intensity}\ast 100$$

Detailed hyper-parameter tuning procedure for the number of trees and features is shown in supplementary information (Supplementary Figure S2 and Supplementary Figure S3).

### Outcome variable: Binary shock index

The SAFE-ICU initiative^8^ developed and described earlier has warehoused over 3,00,000 patient-hours of monitoring data from the PICU and was used to extract time-stamped data for corresponding patients at 0 hr, 3 hr, 6 hr and 12 hr blood pressure and heart rate recordings. Shock index was calculated as the ratio of median heart rate and median arterial systolic blood pressure (both calculated over 30-minute straddling windows). Finally, age-specific binarized outcome (shock/no-shock) was computed for each patient using shock-index pediatric age-adjusted (SIPA) guideline^24^.

### Prediction time-points

The time-point for shock-detection model was 0 hr whereas and the time-points for shock-prediction models were 3 hr, 6 hr, 12 hr from the time of imaging.

### Modeling for shock detection and prediction

Generalized linear mixed effects (glmer) models^25^ were constructed with Bound Optimization By Quadratic Approximation^26^ (bobyqa). The library lme4^27^ in R^28^ (version 3.4.3) was used. Age and pulse rate were included as covariates in the glmer model.

### Model Evaluation

Repeated data were first partitioned patient-wise using patient IDs to avoid cross-contamination between training and test sets. Ten random partitions with 70% training and 30% testing sets respectively were then created for evaluation of stability and accuracy of the model using the caret^29^ library in R^28^. Model performance was assessed using Area under the curve in the receiver operating curve (ROCs) (AUROC). The standard measures of sensitivity, specificity, positive predictive value and negative predictive value were obtained by taking threshold as Youden’s Index, *J* = max(*Se*(*c*) + *Sp*(*c*) − 1)^30^.

## Electronic supplementary material

Supplementary Info

## Data Availability

All our data are made available on Open Source Framework (DOI 10.17605/OSF.IO/VP86J) and the code for modeling is open-sourced on GitHub and Zenodo (https://doi.org/10.5281/zenodo.1256486).
