# Supplementary material for: Predicting Hemodynamic Shock from Thermal Images using Machine Learning
Source: Sci Rep. 2019 Jan 14;9:91. doi: 10.1038/s41598-018-36586-8 (PMC6331545; doi:10.1038/s41598-018-36586-8)
Supplement: Supplementary file 1 — Supplementary Info [file 41598_2018_36586_MOESM1_ESM.pdf]

Supplementary Information

Predicting Hemodynamic Shock from Thermal Images using Machine Learning

Aditya Nagori[1,2], Lovedeep Singh Dhingra[3], Ambika Bhatnagar[3], Rakesh Lodha[3], TavpriteshSethi[*3,4,5]

1. CSIR-Institute of Genomics and Integrative Biology, New Delhi, 110007, India
2. Academy of Scientific and Innovative Research (AcSIR),Ghaziabad, 201002, India
3. All India Institute of Medical Sciences, Department of Pediatrics, New Delhi, 110029, India.
4. Indraprastha Institute of Information Technology Delhi, 110020, Delhi, India
5. Stanford University, School of Medicine, Stanford, 94305, CA, USA

*Corresponding author: Dr. Tavpritesh Sethi, MBBS, PhD, tavpriteshsethi@iiitd.ac.in

Additional Information: *This work was supported by the Wellcome Trust/DBT India Alliance Fellowship IA/CPHE/14/1/501504 awarded to Tavpritesh Sethi.*

Disclosure of competing interests: The authors declare that there are no competing interests.

I. **Supplementary Figure S1**. Comparison of AUCs at different time-points of prediction for manual and automated methods.

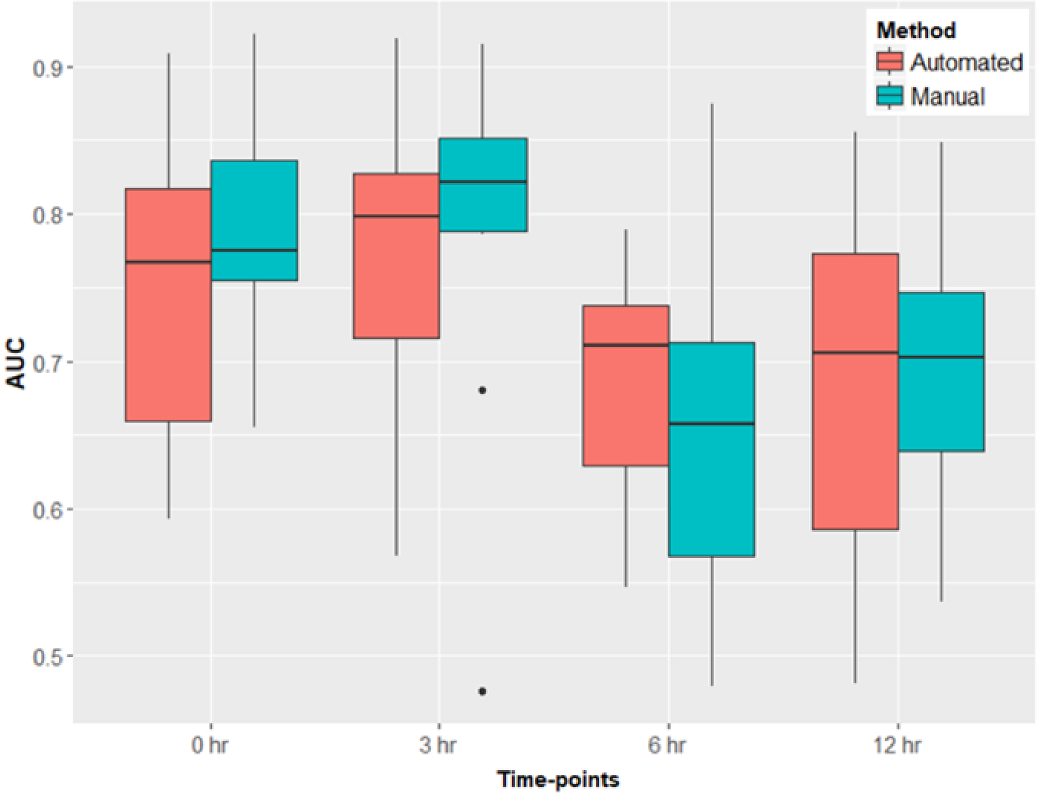

Supplementary Figure S1. The manual and automated pipelines followed each other closely with manual performing slightly better at 0hr and 3hr whereas the automated performing for prediction of shock at 6hr and 12hr respectively. The whiskers represent the spread of AUC values obtained across the ten folds of cross-validation.

II. **Supplementary Figure S2.** Hyper–parameter tuning for **abdomen** classifier using random forests.

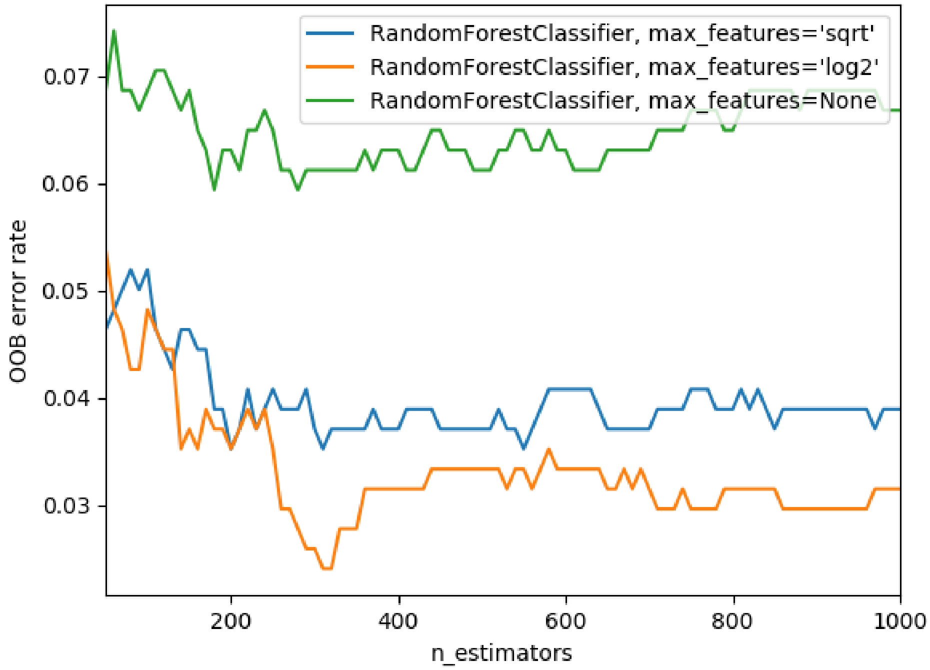

Supplementary Figure S2. Tuning of n-estimators (i.e. the number of trees) and max features done for **abdomen** classifier. The RF model was optimized for these parameters through evaluation of out-of-bag (*OOB*) error with increase in *n-estimators* and the choice of *max-features* between *square-root* and *log2* of the number of features. Minimum OOB was obtained at 310 *n-estimators* and *log2* of number of features which were taken as optimized hyper-parameters for abdomen classifier.

III. Supplementary Figure S3. Hyper–parameter tuning for **foot** classifier using random forests.

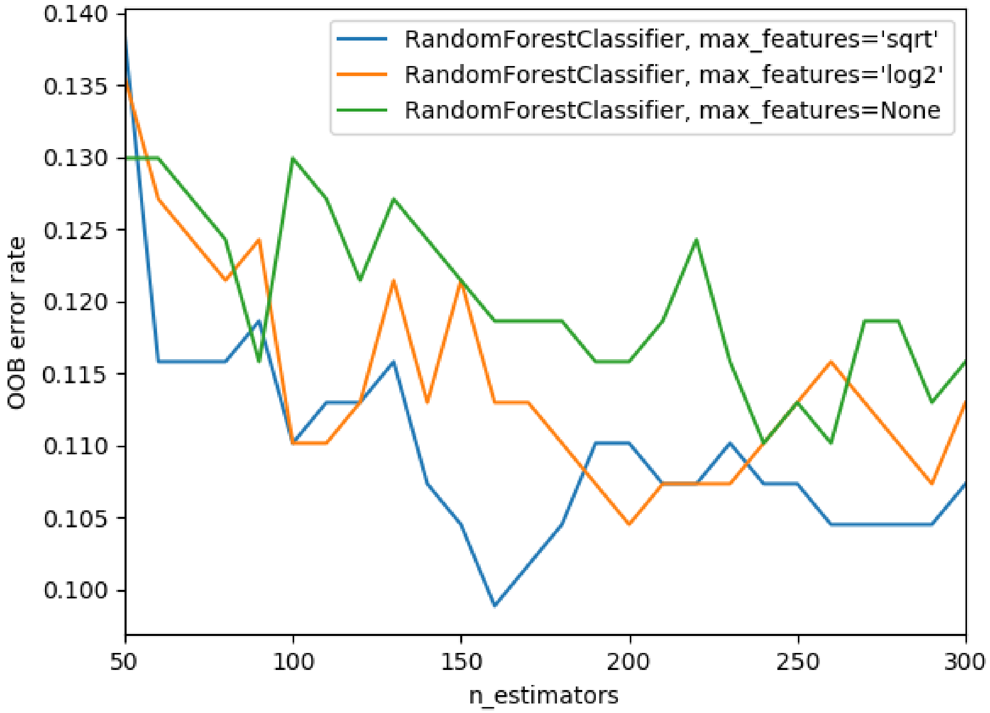

Supplementary Figure S3. Tuning of n-estimators (i.e. the number of trees) and max features done for **foot** classifier. The RF model was optimized for these parameters through evaluation of out-of-bag (*OOB*) error with increase in *n-estimators* and the choice of *max-features* between *square-root* and *log2* of the number of features. Minimum OOB was obtained at 160 *n-estimators* and *square-root* of number of features which were taken as optimized hyper-parameters for foot classifier.

IV. Supplementary Figure S4. Receiver operating characteristics for prediction of shock using **manually derived** CPD.

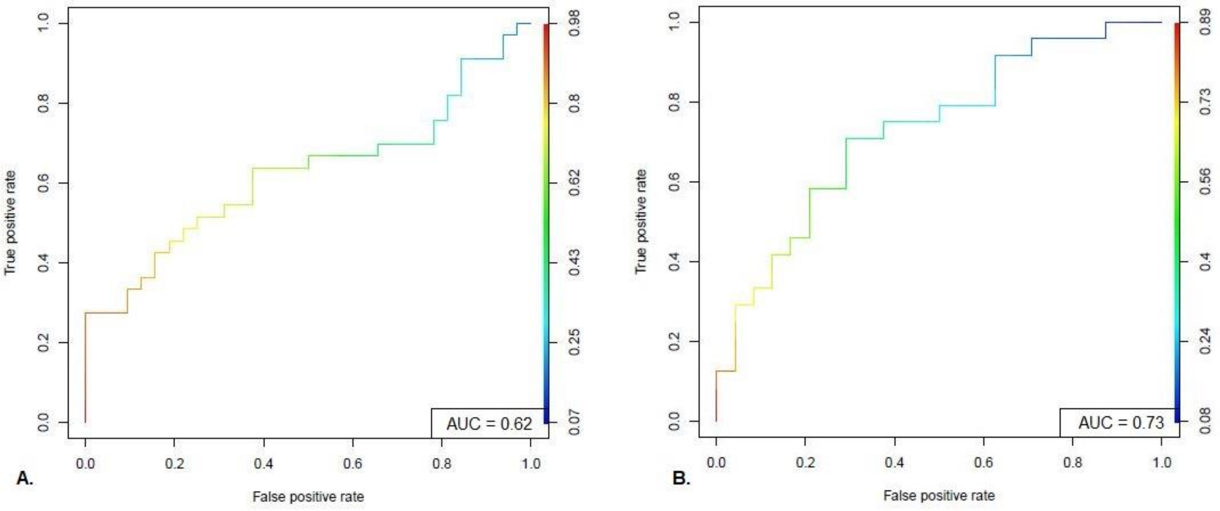

Supplementary Figure S4: Receiver Operating characteristics (ROC) curves for prediction of binary shock-index post 6hr (A) and 12hr (B) of imaging using **manual** CPD. The ROC curves shown are the ones closest to the mean ROC (64% and 70%) obtained through ten random partitions with 70% training and 30% testing sets at 6hr (A) and 12 hr (B) respectively.

V. **Supplementary Figure S5.** Receiver operating characteristics for prediction of shock using **automated** CPD.

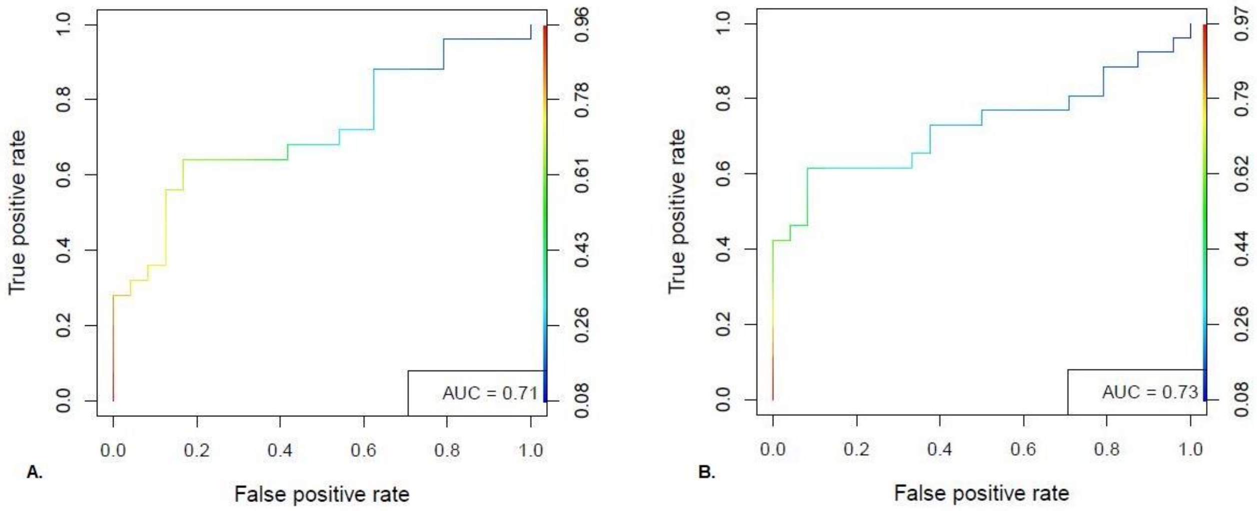

Supplementary Figure S5: Receiver Operating characteristics (ROC) curves for prediction of binary shock-index post 6hr (A) and 12hr (B) of imaging using **automated** CPD. The ROC curves shown are the ones closest to the mean ROC (68% and 69%) obtained through ten random partitions with 70% training and 30% testing sets at 6hr (A) and 12 hr (B) respectively.

VI. **Supplementary Table S1**: Modeling the relationship between Intra-arterial (Invasive) and Non-invasive BP.

	**Value**	**Std.Error**	**DF**	**t-value**	**p-value**
**(Intercept)**	76.58295	7.545633	285	10.14931	0
**NIBP Sys**	0.16615	0.070766	285	2.34786	0.0196

Supplementary Table S1. Estimated fixed-effects of the model where ABP is taken as a function of NIBP. We evaluated whether NIBP can be used as a surrogate for ABP via a reliable relationship that could possibly be drawn between these. Mixed effects regression model was built to account for longitudinal data. Positive coefficient of NIBP shows that for a given value of NIBP we have a higher value of Arterial Blood pressure. However as mentioned in the main manuscript the goodness of fit estimator (R-squared) on testing set was only 33% implying that a reliable relationship could not be drawn from NIBP. Hence we limited ourselves to the set of patients with ABP recordings.

VII. Supplementary Methods S1. Standard operating protocol (SOP) for capturing thermal images.

1. Wear shoe covers, scrub and wear a disposable gown before entering ICU.
2. Connect the Seek thermal imaging camera to the smartphone and open the Seek thermal app.
3. Make sure the app is set to “Iron” theme before capturing images and to high resolution setting.
4. For each patient recorded in the session, do the following steps:-
   - i. At the bedside, use a screen to ensure patient privacy. Uncover the patient if covered by sheet. The nurses’ help can be taken at this step. Undergarments were usually not removed unless abdomen wasn’t visible properly. For babies older than 4-5 years of age, nappies need not be removed.
   - ii. Make the patient comfortable
   - iii. Use a selfie-stick or a stool to ensure the whole body is in the frame and click the image. In bigger children/adults, at least the abdomen and feet should be in the same frame.
   - iv. Open the excel sheet for data collection on a tablet and record the data for the identification of the image such as UHID and bed number image name and date time.
   - v. Take back-up of the images from phone to stable storage in to identified folders with patient UHID Bed information and date.
